# US-Guided Biopsies: Overarching Principles

**DOI:** 10.3389/fmed.2019.00001

**Published:** 2019-01-22

**Authors:** Laurent Meric De Bellefon, Ilias Lazarou

**Affiliations:** ^1^Cliniques Universitaires Saint-Luc, Brussels, Belgium; ^2^CHU Saint-Pierre, Brussels, Belgium; ^3^Department of Rheumatology, Université de Genève, Geneva, Switzerland

**Keywords:** synovial biopsy, anesthesia—local, arthritis (including rheumatoid arthritis), antisepsis, ultrasound guided biopsy techniques, local anesthesia

## Abstract

Gathering synovial tissue from any swollen joint especially in early arthritis patients is critical for good quality research and to obtain further insight into the pathophysiology of inflammatory joint diseases. Multiplying biopsy sites is a challenge in terms of the techniques needed for each different joint but also in terms of safety and tolerability. It is important to provide the best care especially in very early arthritis patients who have only had the disease for a few months. This review discusses the minimal requirements applying to antiseptic techniques for the operator's hands, patient preparation, local anesthesia, and post-procedure care.

## Introduction

Synovial biopsy with ultrasound guided techniques is a safe and well-tolerated procedure however it remains invasive. As such, rheumatologists have to follow some basic aseptic techniques in order to avoid complications.

This review will first discuss the preparation of the patient and the operator, then local anesthetics, the possibility of corticosteroid injections, and post-procedure care.

## Asepsis for the Operator

### Surgical Hand Antisepsis

Preoperative cleansing of hands and forearms with an antiseptic agent has been an accepted practice since the late 1800s (1). Despite a large body of indirect evidence for the need of hand antisepsis prior to surgical interventions this has never been proved by randomized, controlled clinical trials.

United States of America (USA) guidelines recommend the use of agents for surgical hand scrubs which substantially reduce microorganisms on intact skin, contain a non-irritating antimicrobial preparation, have broad-spectrum activity, and are fast-acting and persistent (2, 3).

Reducing resident skin flora on the hands of the surgical team for the duration of a procedure reduces the risk of bacteria being released into the surgical field if gloves are punctured or torn during surgery (4–6).

The World Health Organization (WHO) has provided very precise definitions and has also described operator's hand antisepsis step by step (7–9). Surgical handscrubbing refers to the use of soap and water, while surgical handrubbing is the use of a waterless, alcohol-based solution. The alcohol-based (hand) rub is an alcohol-containing preparation (liquid, gel, or foam) designed to be applied to hands to kill microorganisms and/or temporarily suppress their growth.

### Which Products Should be Used for Surgical Hand Preparation?

There are slight differences in terms of requirements between USA and European guidelines. Guidelines in the USA recommend that agents used for surgical hand preparation should significantly reduce microorganisms on intact skin, contain a non-irritating antimicrobial preparation, have broad-spectrum activity, and be fast-acting and persistent (10). In Europe, all products must have at least the same efficacy as a reference surgical rub using n-propanol, as outlined in the European Standard EN 12791. In contrast to the USA guidelines, only the immediate effect after the hand hygiene procedure and the level of regrowth after 3 h under gloved hands are measured. The cumulative effect over 5 days is not an EN 12791 requirement.

Surgical hand antisepsis can be achieved using medicated soap such avec chlorhexidine gluconate (CHG) 4% or povidone-iodine which both result in similar reductions of bacterial counts (70–80%). Despite both *in vitro* and *in vivo* studies demonstrating that povidone-iodine is less efficient than chlorhexidine, it remains one of the widely-used products for surgical hand antisepsis, although it induces more allergic reactions, and does not have similar residual effects (11, 12).

Surgical hand preparation with alcohol-based handrubs seems to be a safer method with a higher reduction of bacterial counts compared to other agents and a greater acceptability and fewer adverse effects on skin. Only alcohol-based hand gels which have passed the EN 12791 test or an equivalent standard for handrub formulations e.g., FDA TFM 1994, should be used (13). Such preparations usually contain 60–95% ethanol or isopropanol.

Both methods are suitable for the prevention of Surgical Site Infections (SSIs) but WHO panel experts have declared a preference for alcohol-based products.

### Key Steps Before Entering the Operating Theater

Rings, wristbands and watches must be removed and nails must be short and clean without nail-polish. False nails should also be avoided.

Hands and forearms may be washed with non-medicated soap and water. This part is not necessary unless hands are visibly soiled or dirty but it is highly recommended to eliminate any risk of colonization with bacterial spores (14–16).

### Aseptic Procedure

Here we describe the alcohol-based handrub (ABHR) according to WHO recommendations. Apart from a few cases of very large hands and forearms, 15 ml of ABHR are usually enough for the whole procedure.

First, fingertips and forearms are cleaned using 5 milliliters—or 3 doses of ABHR—for each side. This takes ~1 min with an emphasis on the forearms. Second, hands are then rubbed with 5 ml of ABHR keeping the hands held higher than the elbows.

The whole handrub procedure lasts 1.5 min with the recommended ABHR formulations.

The operator's hands are then considered sterile and the operator can enter the procedure room and put on the sterile gloves (2 pairs) and gown.

## Material Needed and Technique

### Table Preparation

**Figure 1:**
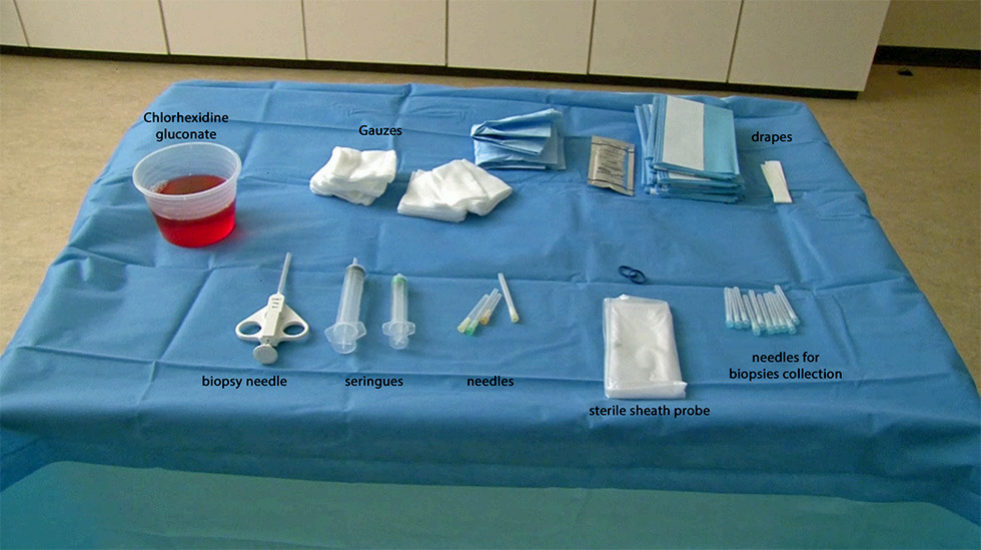
Prepared table with the material needed for ultrasound-guided synovial needle biopsy.

On a sterile drape, place sterile gauzes (5 × 5 or 7.5 × 5 cm), sterile drapes (adhesive 75 × 75 or 140 × 190 non-adhesive), 10–20 needles for biopsy collection, 1 sterile probe sheath, needles for local anesthetic (1 26-G for the skin, 1 20G 50 mm and 1 18G 50 mm), syringes (20 ml for CHG, 10 ml for lidocaine) (Figure 1). The disposable biopsy needle or the instruments portal & forceps (18 G needle, wire, and dilators, optional metallic instruments, flexible, and/or rigid forceps) are also placed on the sterile drape. According to the antimicrobial agents chosen, 150 ml of CHG or PVI-I are usually enough.

The sterile probe sheath may need to have non-sterile ultrasound gel poured inside it to maintain contact with the probe. Some probe sheaths have an adhesive area for the probe so that no gel is needed. As a contact medium between the sheath and skin, we prefer to use chlorhexidine gluconate 4% rather than a sterile gel. A volume of 20 ml is usually sufficient for the whole procedure.

### Ultrasound-Guided Synovial Biopsy Techniques

Ultrasound-guided synovial biopsies can be performed using two different techniques. One uses a portal and forceps (P&F) where a modified Seldinger technique is used to position the coaxial sheath and also to provide a portal for irrigation. The biopsies are then performed with a flexible or rigid forceps under ultrasound guidance (17). The other technique uses a dedicated disposable semi-automatic guillotine-type biopsy needle (BN). It can be used with or without an introducer according to the size of the biopsied joint. Several disposable devices are available, e.g., Tru-Cut (UK Medical) or Quick-Core (Cook Medical), Temno Evolution (BD). There may be some differences found in needle rigidity, the shape of needle bevel, the sensitivity of the semi-automatic mechanism trigger.

The USG biopsies performed either with P&F or a disposable needle offer the same quality of histological analysis of the tissues and the same safety in terms of side effects. Tolerability of both is also good and comparable.

The main differences between these two techniques are that procedure duration is marginally higher for P&F, that BN uses disposable material compared to autoclavable equipment for P&F, and that P&F often requires two operators (18).

## Patient Preparation

### Patient Position and Procedure Room

The patient may be sitting or lying comfortably on a bed according to the target joint. Comfort is particularly important for arthritic patients with active disease.

The patient has to remove rings and bracelets, and has to wear a mask. Shaving of the area where the needle is to be introduced is not required.

Enough space must be provided for the ultrasound machine on the side of the patient opposite the operator, and for the sterile gown to be put on with the aid of an assistant. A dedicated room for the procedure is recommended but any space fulfilling local patient safety standards may be used.

### Patient Asepsis

The aim of this procedure is to reduce the microbial load on the patient's skin as much as possible before breaking the skin barrier.

In Europe, the antimicrobial agent recommended is chlorhexidine gluconate 4% (CHG) but povidone-iodine is also frequently used. In the USA, despite the fact that chlorhexidine gluconate is superior to povidone-iodine for patient preoperative skin preparation, it is still not eligible for that use because of different standards for efficacy.

CHG is a cationic bisbiguanide developed in England in the early 1950s. It is effective on Gram positive and negative bacteria and also against lipophilic viruses (Human Immunodeficiency Virus, influenza, herpes simplex). It has a persistent antimicrobial action that prevents regrowth of microorganisms for up to 6 h. There is no evidence of CHG being toxic if it is absorbed through the skin. This point is crucial and explains why sterile gels are not essential as a contact medium during the ultrasound procedure, as they can be replaced by CHG. Finally, there is a low incidence of hypersensitivity reactions and skin irritation but one has to keep in mind that some severe allergic reactions have been reported (including anaphylaxis).

Povidone-iodine contains 9–12% available iodine and is eligible for patient antiseptic skin preparation, health care personnel hand washing and surgical hand scrubbing. Bacteria do not develop resistance to PVP-I (19).

**Figure 2:**
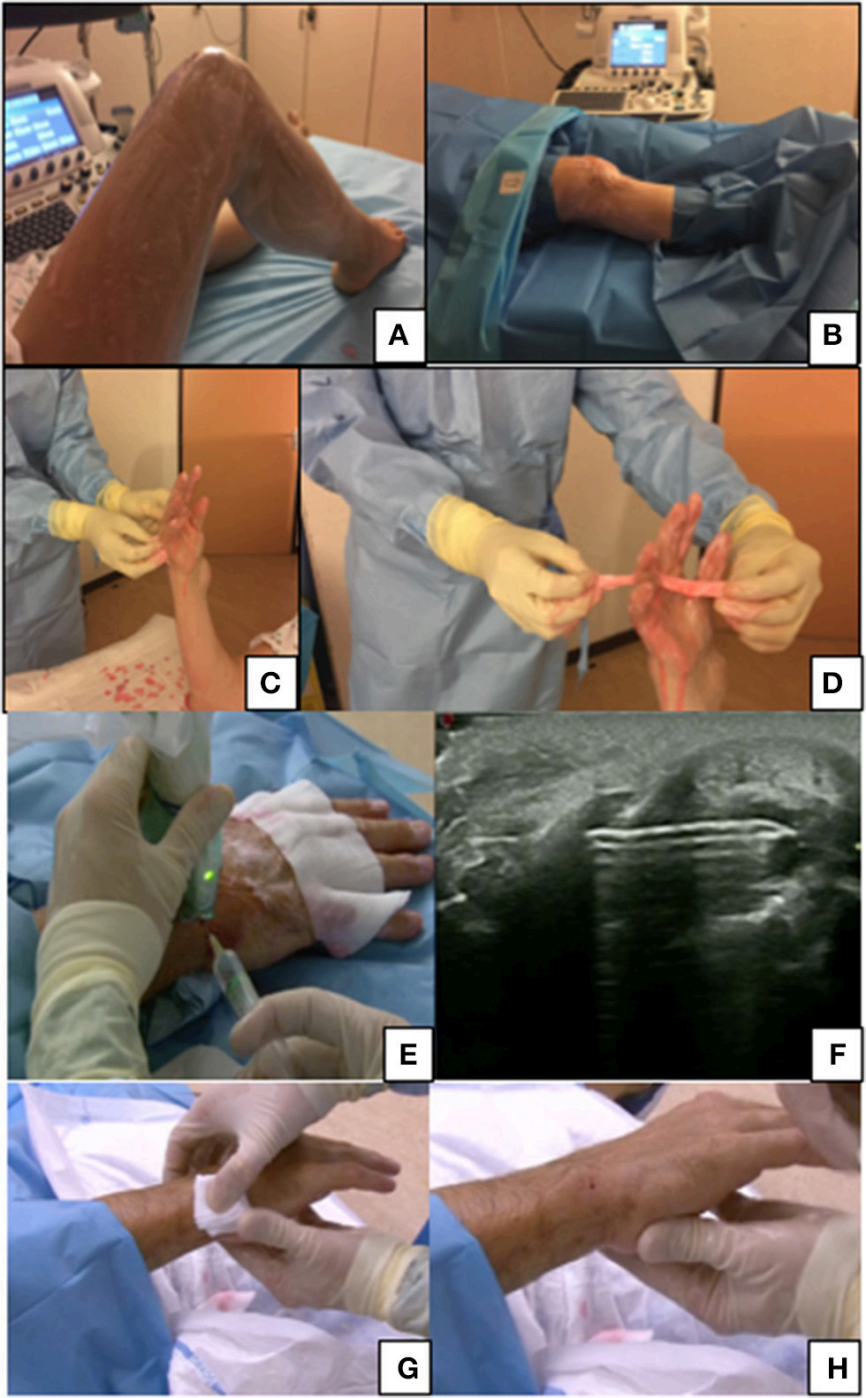
**(A,B)** Preparation for knee biopsy, patient lying. **(C,D)** Hand washing before a biopsy of wrist/PIP with a focus on the interdigit region washing. **(E,F)** Articular anesthesia after skin anesthesia with 18G 50 mm needle under ultrasound guidance. **(G,H)** Post-procedure care, a 2 min compression of the hole entrance before putting a small dressing.

The area to wash will obviously depend on the target joint:

- For the wrists, metacarpophalangeal (MCP), and proximal interphalangeal (PIP) joints: hand and forearm up to the elbow (Figures 2C,D).
- For the elbow: most of the arm and forearm excluding the distal third of the forearm and proximal third of the arm
- For the knee; most of the thigh and the leg excluding the distal third of the leg and proximal third of the thigh (Figures 2A,B).
- For the ankles, metatarsophalangeal (MTP), and proximal interphalangeal (PIP) joints: the leg up to the knee, the ankle and foot.

The area of interest is washed with sterile compresses dipped in the chosen antimicrobial agent solution (CHG or PVP-I). A couple of rubbings of the area of interest are usually enough. For hands and feet, special care should be taken for the interdigital areas and nails. After disinfecting, sterile drapes should be placed to isolate the target joint.

The first pair of gloves (no longer considered sterile) should be discarded.

The assistant (e.g., nurse) has to wear a mask and gloves. One member of the family may be present according to local policies, and they should also wear a mask.

## Local Anesthesic

Lidocaine 1% (w/v) is recommended for the local anaesthesic (LA). The volume depends on the size of the target joint from 3 ml for MCP and PIP to 10 ml for big joints such as knees. The maximal dose is 4–5 mg/kg. In adults, the average lidocaine injected dose is far below the maximal dose. For example, in a 60 kg patient, injection should not exceed 300 mg of lidocaine, whereas 10 ml of lidocaine 1% (10 mg/ml) only correspond to 100 mg. Doses must be carefully calculated for children.

The LA is performed under ultrasound guidance with a suitable needle from the skin to the synovial hypertrophy and the anesthetic effect is usually very quick (1–3 min). Alternatively, a subcutaneous needle can be used for the skin followed by the deeper injection with a thicker needle (Figures 2E,F).

### Adverse Events

Side effects—neurological and cardiovascular—are more common in cases of overdosing or intravascular injection. Patients may experience paraesthesia, a metallic taste, blurred vision, tinnitus, an increase in blood pressure or cardiac arrhythmias.

### Chondrotoxicity of Anesthetic Agents

Severe cartilage damage has been reported with the use of local anesthetic but mainly with continuous intra-articular infusion with bupivacaine, the gleno-humeral joint being the most commonly affected. Thus far, there is no clinical evidence of chondrolysis resulting from a single injection of local anesthetic but rheumatologists have to be aware that *ex-vivo* studies have demonstrated that bupivacaine, lidocaine, ropivacaine, and levobupivacaine are toxic for cartilage. The mechanisms are still unknown, but mitochondrial DNA damage or chemical incompatibility have been suggested and there seems to be a dose- and dose-over-time effect on toxicity (20, 21).

In animal models the assessment of *in vitro* chondrotoxicity showed a dose- and time-dependent effect of lidocaine on the viability of articular cells (22, 23).

### Antimicrobial Effect of Lidocaine

Lidocaine like the other local anesthetic agents possesses bacteriostatic, bactericidal, fungistatic, and fungicidal properties. This role has been documented with *in vitro* and *in vivo* studies since 1950. The exact mode of action is not known but some believe that local anesthetics cause a disruption of microbial cell membrane permeability, leading to a leakage of cellular components and subsequent cell lysis. Lidocaine demonstrated a dose-dependent inhibition of growth for all strains of bacteria tested, with the most activity against gram-negative organisms, and the least against *Staphylococcus aureus*. The addition of epinephrine to the local anesthetic had no effect on the susceptibility of the bacteria to lidocaine.

Thus, on the one hand lidocaine is beneficial in preventing joint infections after invasive procedures, but on the other hand, it could lead to false-negative results or suboptimal culture yields for biopsies (24, 25).

## Post-procedure Care

Theoretically ultrasound guided synovial biopsy procedure may cause infection, bleeding, or lesions of tendons or nerves. This is why the ultrasound pre-biopsy assessment is important, with the identification of the vascular structures and the tendons in the joint of interest. The continuous visualization of the needle and its tip throughout the procedure is also important for the same reason. This is important for complex joint such as wrists, elbows, or ankles biopsies. In the event of unexpected bleeding, clinical examination, and surveillance is recommended.

The very good tolerability of the ultrasound guided synovial biopsy has been demonstrated in many studies and no intense pain should be expected at short- or long-term after biopsies (26).

In practice, once the procedure is finished, the entry site is gentle cleansed with sterile water. A small dressing is placed after 1–2 min of compression on the entry site where there should be a tiny red spot (Figures 2G,H). A bandage can be put around the biopsied joint but is not essential. The dressing and the bandage can be removed the next morning.

Contact details of the operator or department should be given to the patient in case of significant pain, swelling, bleeding, or neurologic symptoms during the week after the procedure.

## Intra-articular Glucocorticoid (GC) Injections

Methylprednisolone 40 mg/ml, triamcinolone acetonide 40 mg/ml or triamcinolone hexacetonide 20 mg/ml can be injected into the joint under ultrasound guidance of the at the end of the biopsy procedure if needed. A 50 mm long 20G needle is suitable for the injection. With the exception of large joints, some of the GC might leak from the needle entry site since pressure builds up during the procedure and LA injection.

In terms of safety, intra-articular glucocorticoid injections are safe with a low incidence of septic arthritis: 1/27.000 in a Dutch retrospective study from 2008 and 2013 (27). In a retrospective multicentric study in patients undergoing synovial biopsies using different techniques (ultrasound-guided or arthroscopic-guided), Soeren et al. recently reported 38 intra-articular joint injections without any increase in adverse events including infections. They were also associated with a statistically significant reduction in post biopsy swelling (28).

## Conclusions

Aseptic techniques for preoperative preparation of patient's skin may vary slightly according to your country or your hospital but their basis and definitions are precise and based on numerous studies. Every rheumatologist who starts performing synovial biopsies has to refresh or acquire knowledge in this specific domain. To date, precise, validated and easily accessible recommendations are published. USG biopsies either with P&F of with a disposable needle biopsy require heeding these precautions. Chlorhexidine gluconate and povidone-iodine can be used for patient skin preparation while alcohol-based handrubs are used for surgical hand preparation.

One has to be aware of the maximal dose, side effects and potential chondrotoxicity of local anesthetics. Ultrasound pre-biopsy examination is important so as to choose the joint of interest and to assess biopsy feasibility. Identification of the different structures (tendons, blood vessels, nerves) along the needle path toward the synovial thickening prevents many problems.

Finally, intra-articular glucocorticoid injections can be safely performed at the end of the procedure if clinically necessary.

## Author Contributions

All authors listed have made a substantial, direct and intellectual contribution to the work, and approved it for publication.

### Conflict of Interest Statement

The authors declare that the research was conducted in the absence of any commercial or financial relationships that could be construed as a potential conflict of interest.
